# Hypertriglyceridaemia as a risk factor for critical care admission in acute pancreatitis: A prospective study

**DOI:** 10.1016/j.clnesp.2020.06.008

**Published:** 2020-10

**Authors:** Alfred Adiamah, Anisa Kushairi, Sue Tumilty, Yuuki Na, Martin Crook, Adam J. Brooks, Dileep N. Lobo, Nottingham University Hospitals Hepatopancreaticobiliary team, E. Alabraba, I.J. Beckingham, I.C. Cameron, D. Gomez, G.R. Irving, A. Koh, A.P. Navarro, E. Psaltis, M. Shaw

**Affiliations:** a Gastrointestinal Surgery, Nottingham Digestive Diseases Centre and National Institute for Health Research (NIHR) Nottingham Biomedical Research Centre, Nottingham University Hospitals NHS Trust and University of Nottingham, Queen's Medical Centre, Nottingham, NG7 2UH, UK; b Department of Clinical Biochemistry, Guy's and St. Thomas' NHS Foundation Trust, London, SE1 9RT, UK; c MRC Versus Arthritis Centre for Musculoskeletal Ageing Research, School of Life Sciences, University of Nottingham, Queen's Medical Centre, Nottingham, NG7 2UH, UK

**Keywords:** Hypertriglyceridaemia, Acute pancreatitis, Aetiology, Clinical outcomes

## Abstract

**Background and aims:**

Hypertriglyceridaemia is both a primary cause of acute pancreatitis and an epiphenomenon. This study aimed to define the associations between hypertriglyceridaemia and clinical outcomes in patients admitted with acute pancreatitis.

**Methods:**

This single-centre prospective observational study included patients with a confirmed clinical, biochemical or radiological diagnosis of acute pancreatitis from August 2017 to September 2018. Baseline demographics, aetiology of pancreatitis, and fasting triglyceride concentrations were recorded and assessed against the surrogate markers of severity: admission to critical care, length of stay (LOS), readmission to hospital, and mortality.

**Results:**

In total, 304 patients with a mean ± SD age of 56.1 ± 19.7 years met the inclusion criteria. There were 217 (71.4%) patients with normotriglyceridaemia (<150 mg/dL or <1.7 mmol/L), 47 (15.5%) with mild hypertriglyceridaemia (150–199 mg/dL or 1.7–2.25 mmol/L) and 40 (13.2%) with moderate-to-severe hypertriglyceridaemia (≥200 mg/dL or >2.25 mmol/L). The underlying aetiologies of acute pancreatitis were gallstones (55%), alcohol (18%), idiopathic (15%), hypertriglyceridaemia (9%), iatrogenic (2%) and bile duct abnormalities (1%). Patients with hypertriglyceridaemia were younger than those with normotriglyceridaemia (p < 0.05). On multivariate regression, moderate-to-severe hypertriglyceridaemia (OR 5.66, 95% CI: 1.87 to 17.19, p = 0.002) and an elevated C-reactive protein concentration ≥120 mg/L (OR 1.00, 95% CI: 1.00–1.01, p = 0.040) were associated with admission to critical care. Moderate-to-severe hypertriglyceridaemia was also associated with an increased LOS (p = 0.002) but not readmission (p = 0.752) or mortality (p = 0.069).

**Conclusion:**

Moderate-to-severe hypertriglyceridaemia in all aetiological causes of acute pancreatitis was predictive of admission to critical care and prolonged LOS but not readmission or mortality.

## 1 Introduction

Acute pancreatitis is a common presentation in hospitals worldwide, with an incidence of 70 cases per 100,000 population per annum [1]. It involves sudden inflammation of the pancreas and can lead to local and systemic complications. While gallstones and alcohol are the most common causes of acute pancreatitis, hypertriglyceridaemia is reportedly the third commonest cause [2]. Hypertriglyceridaemia is defined as serum triglyceride concentration of more than 150 mg/dL or 1.7 mmol/L and is present in 31% of the adult population in the United States [3]. Severe hypertriglyceridaemia refers to serum concentrations ≥1000 mg/dL (11.3 mmol/L) and 15–20% of patients identified as having severe hypertriglyceridaemia develop acute pancreatitis [2].

Some studies have reported that regardless of the underlying primary aetiology of acute pancreatitis, concurrent hypertriglyceridaemia is associated with greater severity, more frequent development of persistent organ failure and poorer outcomes [4]. Others have suggested that hypertriglyceridaemia is associated with aggravation of acute pancreatitis, increased local and systemic complications, and increased admission to ICU [5,6]. A single centre study of 50 patients with severe hypertriglyceridaemia and acute pancreatitis described a mortality of 15% in such patients [7]. Deng et al. found that on univariate analysis of patients with severe acute pancreatitis, those with hypertriglyceridaemia had a significant risk of mortality (13.1%) compared with patients with normotriglyceridaemia [8].

The exact pathophysiological mechanism of hypertriglyceridaemia in triggering and or exacerbating acute pancreatitis is still debatable. The most widely considered theory proposed by Havel [9] suggests that occlusion of pancreatic capillaries by chylomicrons leads to local hydrolysis of these triglyceride-rich lipoproteins and release of high local concentrations of free fatty acids. These aggregate into micelles with detergent properties that injure the vascular endothelium and acinar cells of the pancreas. The ensuing ischaemia creates an acidic environment, which triggers further free fatty acid toxicity. The cyclical micelle-free fatty acid production and the potential to induce ischaemia and necrosis may, in part, contribute to the suggested severity associated with hypertriglyceridaemia in the context of pancreatitis [9,10]. Other mechanisms suggested include a genetic predisposition and metabolic abnormalities including insulin resistance.

This study aimed to define, using a prospective cohort, the associations between hypertriglyceridaemia and clinical outcomes in patients admitted with acute pancreatitis.

## 2 Methods

### 2.1 Study population

Consecutive patients diagnosed with acute pancreatitis from August 2017 to September 2018 at Nottingham University Hospitals NHS Trust, Queen's Medical Centre in the United Kingdom were included in this prospective study. Acute pancreatitis was diagnosed based on clinical features, biochemistry or imaging: acute onset epigastric pain often radiating to the back and elevated serum lipase concentrations ≥3 times the upper limit of normal, and/or evidence of acute pancreatitis on imaging. The study was registered with and approved by the hospital audit department (Approval no. 17-199c). The need to obtain consent from patients was waived.

### 2.2 Definition and classification of hypertriglyceridaemia

In this study, hypertriglyceridaemia was defined based on guidelines by the American Heart Association, National Lipid Association and The Endocrine Society [3,11,12]. Any patient with serum triglyceride concentrations of ≥150 mg/dL or 1.7 mmol/L was classified to have hypertriglyceridaemia. Furthermore, hypertriglyceridaemic patients were subcategorised into mild hypertriglyceridaemia (150–199 mg/dL or 1.7–2.25 mmol/L) and moderate-to-severe hypertriglyceridaemia (≥200 mg/dL or >2.25 mmol/L) [11].

### 2.3 Definition of primary aetiology

The primary aetiology of acute pancreatitis was determined based on definitions summarised in Table 1. Gallstone pancreatitis was diagnosed when gallstones were detected on ultrasound. Acute pancreatitis secondary to alcohol excess was diagnosed if patients met the criteria for alcohol excess on admission, namely drinking 35 units a week or more for women, and 50 units a week or more for men [13].

**Table 1:** Definition of primary aetiology of acute pancreatitis.

Primary Aetiology	Definition
Gallstones	Diagnosis of gallstones on ultrasound
Alcohol	History of recent alcohol excess
Hypertriglyceridaemia	Mild, moderate or severe hypertriglyceridaemia with no gallstones nor alcohol excess
Iatrogenic	Acute pancreatitis as a result instrumentation of the biliary tree or pancreatic duct
Abnormal bile duct	Acute pancreatitis caused by periampullary cancer
Idiopathic	Absence of gallstones, alcohol excess, hypertriglyceridaemia, iatrogenic causes and malignancy

Hypertriglyceridaemia was diagnosed as the primary aetiology of acute pancreatitis if patients had mild or moderate hypertriglyceridaemia in the absence of other causes. However, if a patient had severe hypertriglyceridaemia, this was the primary cause of acute pancreatitis regardless of the presence of other potential aetiologies. There is currently a lack of minimum diagnostic triglyceride concentration for hypertriglyceridaemia associated acute pancreatitis [14]. Iatrogenic acute pancreatitis was diagnosed if acute pancreatitis was caused by a procedure such as endoscopic retrograde cholangiopancreatography (ERCP). Acute pancreatitis was thought to have been caused by abnormal biliary structure in the presence of bile duct malignancy. Lastly, in absence of all causes mentioned above, acute pancreatitis was categorised as idiopathic.

### 2.4 Data collection

Data were collected using the hospital electronic clinical databases and patient digital health record. Baseline demographic data such as age, gender, body mass index and smoking status were recorded. The underlying aetiology was determined based on alcohol history, presence of gallstones on ultrasound and fasting serum triglyceride concentrations collected within 24 h of admission.

Other clinical and biochemical parameters collected included the Acute Physiology and Chronic Health Evaluation II (APACHE II) score, white cell count, and serum C-reactive protein (CRP), lipase and bilirubin concentrations. Blood samples for fasting triglyceride concentrations were taken on the morning after index admission. The outcome measures of interest were admission to critical care [intensive care unit (ICU) or high dependency unit (HDU)] – as a clinical measure of severity of presentation, length of stay (LOS), readmission to hospital and 30-day mortality. Based on the UK guidelines for the management of acute pancreatitis CRP ≥120 mg/L and APACHE II score ≥9 were prognosticators of severe acute pancreatitis [15].

### 2.4 Statistical methods

Data were analysed using STATA/SE 15 software (StataCorp LLC, College Station, TX, USA). Continuous variables were expressed as mean and standard deviation or median (interquartile range), while categorical variables were represented as percentages. Demographic data of the normotriglyceridaemia and hypertriglyceridaemia groups were analysed using the independent samples *t*-test (for normally distributed variables) and the Mann-Whitney U test (for non-parametric variables). One-way analysis of variance (ANOVA) was used where there were more than two independent groups. Univariate and multivariate logistic regression analyses were used to identify variables predictive of critical care admission, LOS, readmission to hospital, and mortality. A p value of <0.05 was deemed to indicate a statistically significant difference.

## 3 Results

### 3.1 Study population

In total, 304 patients with a mean ± SD age of 56.1 ± 19.7 years met the inclusion criteria: 217 (71.4%) patients were in the normotriglyceridaemia group, 47 (15.5%) patients in the mild hypertriglyceridaemia group and 40 (13.2%) patients were in the moderate-to-severe hypertriglyceridaemia group (Table 2). The mean ± SD age of patients with acute pancreatitis who had hypertriglyceriademia was significantly lower (mild: 41.2 ± 23.7 years and moderate-to-severe: 48.4 ± 15.0 years) compared with those in the normotriglyceridaemia group (57.7 ± 20.4 years, p = 0.027). There was a greater proportion of male patients in the hypertriglyceriademia groups compared with those in the normotriglyceridaemia group (mild: 63.8%, moderate-to-severe: 60.0% *vs.* normotriglyceridaemia: 47.5%, p = 0.04). The percentage of patients who were overweight or obese were comparable in all groups (normotriglyceridaemia: 34.1% *vs.* mild: 27.7% and moderate-to-severe: 37.5%, p = 0.577). There was a greater percentage of smokers in the hypertriglyceriademia groups (mild: 25.5% and moderate-to-severe: 32.5% *vs.* normotriglyceridaemia: 18.0%, p = 0.063).

**Table 2:** Baseline demographic characteristics and outcome measures of patients.

Parameters	Normotriglyceridaemia (N = 217)	Mild hypertriglyceriademia (N = 47)	Moderate-to-severe hypertriglyceriademia (N = 40)	Total (N = 304)	p value
Demographic data					
Age, mean (SD), years	57.8 (20.4)	41.2 (23.7)	48.4 (15.0)	56.07 (19.7)	0.03∗
18–39, n (%)	49 (22.6)	9 (19.2)	15 (37.5)	73 (24.0)	
40–59, n (%)	62 (28.6)	21 (44.7)	15 (37.5)	98 (32.2)	
60–79, n (%)	63 (29.0)	13 (27.7)	9 (22.5)	85 (28.0)	
≥80, n (%)	43 (19.8)	4 (8.5)	1 (2.5)	48 (15.8)	
Gender					0.04∗
Male, n (%)	103 (47.5)	30 (63.8)	24 (60.0)	157 (51.6)	
Female, n (%)	114 (52.5)	17 (36.2)	16 (40.0)	147 (48.4)	
BMI (kg/m^2^)					0.58
<30, n (%)	137 (63.1)	32 (68.1)	25 (62.5)	194 (63.8)	
≥30, n (%)	74 (34.1)	13 (27.7)	15 (37.5)	102 (33.6)	
Unknown, n (%)	6 (2.8)	2 (4.3)	0	8 (2.6)	
Smoking status					0.06
Smoker, n (%)	39 (18.0)	12 (25.5)	13 (32.5)	64 (21.1)	
Non-smoker, n (%)	174 (80.2)	31 (66.0)	27 (67.5)	232 (76.3)	
Unknown, n (%)	4 (1.8)	4 (8.5)	0	8 (2.6)	
Severity					
APACHE II score < 9	78 (35.9)	20 (44.7)	23 (57.5)	121 (39.8%)	0.07
APACHE II score ≥ 9	92 (42.4)	15 (31.9)	8 (20.0)	115 (37.8%)	
Unknown	47 (21.7)	12 (25.5)	9 (22.5)	68 (22.4%)	
Biochemistry					
Triglyceride concentration, median (interquartile range), mmol/L	1 (0.8–1.2)	1.9 (1.8–2.1)	3.65 (2.4–5.9)	1.2 (0.9–1.8)	<0.01∗
C-reactive protein concentration, median (interquartile range), mg/L	21.0 (6.0–122.0)	23.5 (6.0–83.0)	50.5 (19.0–114.0)	24.5 (7.0–115.0)	0.18
White cell count, mean (SD), x10ˆ9 cells/L	13.2 (5.4)	13.96 (5.5)	13.44 (5.6)	13.3 (5.5)	0.35
APACHE II score, mean (SD)	9.6 (4.6)	8.4 (4.59)	7.4 (3.05)	9.1 (4.50)	0.07
Primary aetiology, n (%)					
Gallstones	133 (61.3)	19 (40.0)	14 (35.0)	166 (54.6)	<0.01∗
Alcohol	28 (12.9)	14 (30.0)	12 (30.0)	54 (17.8)	<0.01∗
Hypertriglyceridaemia	0	14 (30.0)	14 (35.0)	28 (9.2)	<0.01∗
Iatrogenic: post-ERCP or cholecystectomy	6 (2.8)	0	0	6 (2.0)	0.30
Abnormal bile duct	4 (1.8)	0	0	4 (1.3)	0.45
Idiopathic	46 (21.2)	0	0	46 (15.1)	0.04∗
Outcome					
Duration of stay, median (interquartile range), days	4 (2.5–7.0)	6 (3.0–11.0)	5 (2.0–10.5)	4 (2.0–8.0)	<0.01∗
Critical care admission, n (%)	16 (7.4)	8 (17.0)	9 (22.5)	33 (10.9)	0.01∗
Hospital readmission, n (%)	23 (10.6)	6 (12.8)	4 (10.0)	33 (10.9)	0.90
Mortality, n (%)	17 (7.8)	2 (4.3)	4 (10.0)	23 (7.6)	0.58

∗ indicates statistically significant differences (p < 0.05).

There was a greater percentage of patients with APACHE II score of ≥9 in the normotriglyceridaemia group compared with the hypertriglyceridaemia groups (p = 0.067). However, 68 patients (22.4%) had unknown APACHE II scores. Those in the moderate-to-severe hypertriglyceridaemia group had a greater median CRP concentration (50.5 mg/L) compared with the mild hypertriglyceridaemia and normotriglyceridaemia groups (23.5 mg/L and 21.0 mg/L respectively). All groups had high mean ± SD white cell counts (normotriglyceridaemia: 13.2 ± 5.4 *vs*. mild: 14.0 ± 5.5 and moderate-to-severe: 13.4 ± 5.6 × 10^9^ cells/L).

### 3.2 Triglyceride concentrations and hypertriglyceridaemia

The overall median (interquartile range) triglyceride concentration was low at 1.2 mmol/L (0.9–1.8 mmol/L). It was 1.9 mmol/L (1.8–2.1 mmol/L) in the mild hypertriglyceridaemia group and 3.65 mmol/L (2.4–5.9 mmol/L) in the moderate-to-severe hypertriglyceridaemia group.

### 3.3 Primary aetiology of acute pancreatitis

Gallstones were the most common aetiology of acute pancreatitis (55%), followed by alcohol (18%), idiopathic (15%) and hyperlipidaemia (9%). Associated aetiologies are described in Table 2.

### 3.4 Length of stay

Patients with acute pancreatitis and hypertriglyceridaemia stayed in hospital for a median of 1–2 days longer than those with normotriglyceridaemia at admission. The median length of stay when triglycerides were normal were 4 days (interquartile range 2.5–7 days), while patients with mild hypertriglyceridaemia stayed for 6 days (interquartile range 3–11 days) and those with moderate-to-severe hypertriglyceridaemia stayed for 5 days (interquartile range 2–10.5 days), p = 0.0043.

### 3.5 Admission to critical care (ICU/HDU)

Univariate and multivariate logistic regression analyses were performed to identify variables that could predict admission to critical care, mortality and readmission to hospital. The univariate and multivariate logistic regression analyses for critical care admission are summarised in Table 3. Based on the univariate logistic regression; patients with acute pancreatitis and mild hypertriglyceridaemia were 2.5 times more likely to be admitted to critical care: odds ratio (OR) 2.58, 95% Confidence Interval (CI) 1.03 to 6.44, p = 0.043. Patients with moderate-to-severe hypertriglyceridaemia and acute pancreatitis were almost four times more likely to need critical care: OR 3.65, 95% CI 1.48 to 8.97, p = 0.005. Other significant predictors of critical care admission on the univariate analysis were presence of gallstones (OR 0.43, 95% CI 0.20 to 0.90, p = 0.026) and CRP concentrations of ≥120 mg/L (OR 1.01, 95% CI 1.00 to 1.02, p = 0.0001). In the multivariate logistic regression analysis, patients with moderate-to-severe hypertriglyceridaemia were six times more likely to be admitted to critical care (OR 5.66, 95% CI 1.87 to 17.19, p = 0.002).

**Table 3:** Logistic regression for critical care admission.

Parameter	Category	Univariate			Multivariate		
		OR	95% CI	p value	OR	95% CI	p value
Gender	Male	0.10	0.03–0.31				
	Female	1.15	0.56–2.37	0.70	2.44	0.94–6.29	0.07
Age (years)	18–39	0.12	0.06–0.26				
	40–59	1.24	0.49–3.18	0.65	0.96	0.31–3.01	0.94
	60–79	0.96	0.35–2.64	0.94	0.42	0.09–1.88	0.256
	≥80	0.54	0.14–2.15	0.38	0.45	0.06–3.12	0.42
Hypertriglyceridaemia	No	0.08	0.05–0.13				
	Mild	2.58	1.03–6.44	0.04∗	2.59	0.79–8.55	0.12
	Moderate-to-severe	3.65	1.48–8.97	0.01∗	5.66	1.87–17.19	<0.01∗
Gallstones	No	0.18	0.11–0.29				
	Yes	0.43	0.20–0.90	0.03∗	0.704	0.26–1.90	0.49
CRP, mg/L	0–119	0.07	0.04–0.12				
	≥120	1.01	1.00–1.02	<0.01∗	1.00	1.00–1.01	0.04∗
APACHE II score	0–9	0.13	0.07–0.23				
	≥9	0.98	0.07–0.23	0.62	1.06	0.92–1.21	0.42

∗ indicates statistically significant differences (p < 0.05).

### 3.6 Mortality

Univariate logistic regression showed that patients aged 80 years or above with acute pancreatitis were eleven times more likely to die than younger patients (OR 11.83, 95% CI 2.51 to 55.74, p = 0.002). Patients with acute pancreatitis secondary to bile duct abnormality in the form of primary or metastatic malignancy were thirteen times more likely to die in univariate analysis: (OR 13.29, 95% CI 1.78 to 99.11, p = 0.012). However, in both univariate and multivariate regression analysis, hypertriglyceridaemia was not predictive of mortality. The univariate and multivariate regression analyses for mortality are summarised in Table 4.

**Table 4:** Logistic regression for mortality.

Parameter	Category	Univariate			Multivariate		
		OR	95% CI	p value	OR	95% CI	p value
Gender	Male	0.26	0.07–0.93				
	Female	0.44	0.18–1.10	0.08	0.74	0.25–2.21	0.59
Age (years)	18–39	0.03	0.01–0.11				
	40–59	1.91	0.36–10.13	0.45	1.03	0.16–6.56	0.98
	60–79	1.75	0.31–9.86	0.52	0.45	0.04–4.67	0.50
	≥80	11.83	2.51–55.74	<0.01∗	3.71	0.37–36.97	0.26
Hypertriglyceridaemia	No	0.09	0.05–0.14	<0.01∗			
	Mild	0.52	0.11–2.34	0.40	0.43	0.05–4.03	0.46
	Moderate-Severe	1.31	0.42–4.11	0.65	4.08	0.89–18.58	0.07
Gallstones	No	0.10	0.07–0.20				
	Yes	0.50	0.21–1.19	0.12	0.62	0.18–2.09	0.44
Duct anomaly	No	0.08	0.05–0.12				
	Yes	13.29	1.78–99.11	0.01∗	6.27	0.41–96.06	0.19
APACHE II score	0–8	0.05	0.02–0.12				
	≥9	1.10	0.99–1.23	0.08	1.00	0.99–1.34	0.07

∗ indicates statistically significant differences (p < 0.05).

### 3.7 Readmission to hospital

The logistic regression analysis for readmission to hospital is summarised in Table 5. Univariate logistic regression analysis revealed that female patients were twice as likely to be readmitted to hospital following an episode of acute pancreatitis (OR 2.34, 95% CI 1.10 to 5.01, p = 0.029). Patients aged 40–59 were also more likely to be readmitted in the univariate analysis (OR 0.32, 95% CI 0.12 to 0.85, p = 0.022). In the multivariate logistic regression analysis, female gender (OR 2.57, 95% CI 1.13 to 5.81, p = 0.024) and age of 40–59 years (OR 0.30, 95% CI: 0.11 to 0.82, p = 0.019) were significant predictors of readmission to hospital. However, the severity of hypertriglyceridaemia did not influence the risk of readmission to hospital.

**Table 5:** Logistic regression for readmission.

Parameter	Category	Univariate			Multivariate		
		OR	95% CI	p value	OR	95% CI	p value
Gender	Male	0.03	0.01–0.12				
	Female	2.34	1.10–5.01	0.03∗	2.57	1.13–5.81	0.02∗
Age (years)	18–39	0.24	0.13–0.42				
	40–59	0.32	0.12–0.85	0.02∗	0.30	0.11–0.82	0.02∗
	60–79	0.50	0.20–1.23	0.13	0.54	0.21–1.38	0.20
	≥80	0.28	0.08–1.04	0.06	0.33	0.09–1.26	0.11
Hypertriglyceridaemia	No	0.12	0.08–0.18	<0.01∗			
	Mild	1.23	0.47–3.22	0.67	1.33	0.48–3.67	0.58
	Moderate-Severe	0.94	0.31–2.87	0.91	0.83	0.26–2.66	0.75
Gallstones	No	0.15	0.09–0.25				
	Yes	0.65	0.32–1.35	0.25	0.48	0.22–1.05	0.07

∗ indicates statistically significant differences (p < 0.05).

## 4 Discussion

### 4.1 Main findings

The main finding this single centre prospective observational study was that moderate-to-severe hypertriglyceriademia in patients with acute pancreatitis was associated with significantly increased risk of critical care (ICU/HDU) admission. On univariate analysis patients with either mild or moderate-to-severe hypertriglyceriademia, irrespective of underlying aetiology of pancreatitis, had an increased risk of critical care admission, with a 4-fold increased risk in those with moderate-to-severe disease. The significance of moderate-to-severe hypertriglyceridaemia as a risk factor for critical care admission remained even after mutually adjusting for age, gender, gallstones and APACHE II score. Patients with both mild and moderate-to-severe hypertriglyceridaemia also had a longer median hospital LOS than those with normotriglyceridaemia (6 *vs.* 4 days). However, hypertriglyceridaemia did not influence the risk of readmission or mortality. Age and biliary structural abnormality were the factors identified that predicted an increased risk of mortality in this group. However, as there were only 4 patients with structural biliary abnormalities, this must be interpreted with caution.

Demographic data showed that patients with hypertriglyceridaemia were significantly younger than those with normotriglyceridaemia and were more likely to be male smokers. The commonest primary aetiology of acute pancreatitis in our study was gallstones, followed by alcohol, idiopathic causes, hypertriglyceridaemia, iatrogenic causes and structural abnormalities of the biliary system. Eighteen years after an initial study on acute pancreatitis in the same geographical region [16], the trends in aetiology of acute pancreatitis remained consistent; gallstones continue to be the leading cause of acute pancreatitis (49% in 2001, 55% in 2018). The occurrence of alcohol excess as a primary aetiology of acute pancreatitis increased by 3% from 2001 to 2018, consistent with the increasing trend of alcohol use disorders.

### 4.2 What is available in the literature?

Whilst attempts have been made to explore the pathophysiology of hypertriglyceriademia as a primary cause of acute pancreatitis, its role as an epiphenomenon [2] of the condition is less well understood. In this study hypertriglyceriademia was present in 33 of 166 patients who had gallstones (19.9%) and 26 of 54 patients with a history of alcohol excess (48.1%). Alcohol excess, obesity and hereditary disorders have been postulated to lead to secondary hypertriglyceriademia due to increased concentrations of chylomicrons and very-low density lipoproteins (VLDL). Obesity is further thought to exacerbate the severity of acute pancreatitis via effects on cytokines, adipokines, damage-associated molecular patterns and unsaturated fatty acid-mediated lipotoxicity [17]. In the setting of hypertriglyceriademia and another aetiological cause of acute pancreatitis it is difficult to decipher which is an epiphenomenon and which is the primary precipitant of the condition. It is, however, more probable that underlying genetic predilection, metabolic imbalances and environmental factors interact and contribute to the complexity of hypertriglyceriademia within the context of acute pancreatitis.

In the present study, patients with hypertriglyceriademia developed acute pancreatitis at a significantly younger age compared with the normotriglyceridaemic group, consistent with the results of a previous study [4]. Our female patients were twice as likely to be readmitted to hospital after an episode of acute pancreatitis compared with men, before and after adjusting for age, serum triglyceride concentrations and presence of gallstones. Few studies linked gender to outcomes after acute pancreatitis, but it has been observed that being young and male, with a history of alcohol excess were risk factors associated with readmissions after acute pancreatitis [18].

The association between hypertriglyceridaemia in acute pancreatitis and critical care admission is consistent with existing literature. A retrospective observational study of 582 patients with acute pancreatitis in 2016 found that a serum triglyceride concentration ≥2.26 mmol/L in acute pancreatitis was an independent predictor of admission to ICU, and developing local and systemic complications [5]. Similarly, in a large retrospective study of 1539 patients with acute pancreatitis, hypertriglyceridaemia was associated with greater severity and poorer prognosis of acute pancreatitis, persistent systemic inflammatory response syndrome and increased ICU admission [6]. Increased need for ICU admission and an increased overall LOS have also been reported previously [5].

Mortality from acute pancreatitis in our study was 7%, comparable with a local estimate of 7.7% from our previous study [16] and global estimates of 1–7% [19]. On univariate analysis, octogenarians were eleven times more likely to die from acute pancreatitis than any other age group. Univariate and mutually adjusted multivariate analyses demonstrated that patients with primary cholangiocarcinoma or a metastatic biliary lesion causing abnormality in biliary architecture were at significant risk of dying from acute pancreatitis. A meta-analysis performed in 2010 found that the risk of death from acute pancreatitis increased with age, co-morbidities and severity of disease; mortality was highest among patients with organ failure and infected necrosis [20].

### 4.3 Strengths and limitations

Our study comprehensively analysed the occurrence of hypertriglyceridaemia both as a primary aetiology of acute pancreatitis, and as a concurrent phenomenon in other primary aetiologies of acute pancreatitis. We reinforced current evidence on hypertriglyceridaemia and poorer outcomes after acute pancreatitis, particularly with regards to critical care admission and LOS. We were able to compare the present primary aetiology of acute pancreatitis in our geographical region with the primary aetiology in 2001. Moreover, we also shed light on the association between hypertriglyceridaemia and developing acute pancreatitis at a younger age.

However, there are some limitations to our study that need highlighting. The study population was limited to a single centre; experience at a large tertiary teaching hospital and the regional population may not be completely representative. The absence of a sample size calculation may have caused a lack of power to detect a difference in hypertriglyceridaemia and the risk of mortality and risk of readmission. The UK clinical guidelines for the management of acute pancreatitis recommends an APACHE II score of ≥9 as an accurate severity stratification for acute pancreatitis [15]. A significant proportion of missing data on APACHE II scores of patients prevented any meaningful interpretation of the APACHE II scores between patients as a predictor of severity.

There is currently a lack of consensus for the minimum diagnostic serum triglyceride concentration to identify hypertriglyceridaemia as a primary aetiology of acute pancreatitis. In our study, hypertriglyceridaemia was diagnosed as the primary aetiology if patients had mild or moderate hypertriglyceridaemia in the absence of other causes. However, if a patient had severe hypertriglyceriademia, this was defined as the primary cause of acute pancreatitis regardless of the presence of other potential aetiologies. While the presence of severe hypertriglyceridaemia (>11.3 mmol/L, >1000 mg/dL) was considered diagnostic for hypertriglyceridaemia as the primary aetiology [21], triglyceride concentrations in our patients were generally low; only a third of patients had hypertriglyceridaemia. Another study that evaluated hypertriglyceridaemia in acute pancreatitis similarly found elevated triglycerides in a third of the included 1233 patients in agreement with our findings, which consisted of 10% with moderate hypertriglyceridaemia and 22% with severe hypertriglyceridaemia [14]. As there is no clear threshold at which hypertriglyceridaemia is known to trigger acute pancreatitis [10], hypertriglyceridaemia remains a probable primary aetiology of acute pancreatitis if serum triglyceride concentrations are raised and other aetiologies of acute pancreatitis are excluded [21].

## 5 Conclusion

In this study moderate-to-severe hypertriglyceriademia was predictive of critical care admission and increased LOS. Whilst this is suggestive of severity associated with hypertriglyceriademia the lack of an association with mortality is possibly due to a lack of power to detect this. A larger multicentre study to assess critical care admission and mortality with adequate power may be needed in the future to further explore the role of hypertriglyceriademia in the prognostication of acute pancreatitis.

## Funding

This work was supported by the 10.13039/501100000265Medical Research Council [grant number MR/K00414X/1]; and 10.13039/501100000341Arthritis Research UK [grant number 19891]. AA was funded by a 10.13039/100012411National Institute for Health Research (NIHR) Academic Clinical Fellowship. The funders has no role in the design or conduct of the work, or in the decision to publish. The views expressed are those of the authors and not necessarily those of the MRC, ARUK, NHS, the NIHR or the Department of Health.

## Author contributions

Study design: AA, AJB, MC, DNL.

Data collection: AA, AK, ST, YN.

Data analysis: AA, AK.

Writing of the manuscript: AA, AK, YC, MC, DNL.

Critical revision: AA, AK, MC, AJB, DNL.

Final approval: AA, AK, ST, YN, MC, AJB, DNL.

## Declaration of Competing Interests

None of the authors has a direct conflict of interest to declare. DNL has received an unrestricted research grants for unrelated work from B. Braun in the last 3 years. He has also received speakers' honoraria from B. Braun, Fresenius Kabi, Shire and Baxter Healthcare for unrelated work in the last 3 years. AJB has received an unrestricted research grant for unrelated work from Haemonetics Inc. in the last 3 years. He has also received speakers’ honoraria from Haemonetics Inc. and Johnson & Johnson in the last 3 years.

## Appendix A

**Nottingham University Hospitals Hepatopancreaticobiliary team:** E Alabraba, IJ Beckingham, IC Cameron, D Gomez, GR Irving, A Koh, AP Navarro, E Psaltis and M Shaw.
